# Translational Aspects of Diet and Non-Alcoholic Fatty Liver Disease

**DOI:** 10.3390/nu9101077

**Published:** 2017-09-28

**Authors:** Nicolas Goossens, François R. Jornayvaz

**Affiliations:** 1 Division of Gastroenterology and Hepatology, Geneva University Hospital, Rue Gabrielle-Perret-Gentil 4, 1205 Geneva, Switzerland; nicolas.goossens@hcuge.ch; 2 Service of Endocrinology, Diabetes, Hypertension and Nutrition, Geneva University Hospital, Rue Gabrielle-Perret-Gentil 4, 1205 Geneva, Switzerland

**Keywords:** NAFLD, NASH, diet, metabolic syndrome, insulin resistance

## Abstract

Non-alcoholic fatty liver disease (NAFLD) is a spectrum of diseases ranging from simple steatosis without inflammation or fibrosis to nonalcoholic steatohepatitis (NASH). Despite the strong association between dietary factors and NAFLD, no dietary animal model of NAFLD fully recapitulates the complex metabolic and histological phenotype of the disease, although recent models show promise. Although animal models have significantly contributed to our understanding of human diseases, they have been less successful in accurate translation to predict effective treatment strategies. We discuss strategies to overcome this challenge, in particular the adoption of big data approaches combining clinical phenotype, genomic heterogeneity, transcriptomics, and metabolomics changes to identify the ideal NAFLD animal model for a given scientific question or to test a given drug. We conclude by noting that novel big data approaches may help to bridge the translational gap for selecting dietary models of NAFLD.

## 1. Introduction

Non-alcoholic fatty liver disease (NAFLD) affects between 25% to 30% of the general population and is an emerging non-communicable health epidemic [1]. NAFLD encompasses a spectrum of diseases ranging from simple steatosis without inflammation or fibrosis to nonalcoholic steatohepatitis (NASH). Histologically, the disease is characterized by the presence of liver steatosis, hepatocyte ballooning, and inflammation associated with a varying degree of fibrosis [2]. The presence of NAFLD is associated with an increased risk of mortality due to its strong association with type 2 diabetes (T2DM) and other features of the metabolic syndrome [1]. In addition, the presence of NASH, in particular in the context of liver fibrosis or cirrhosis, is associated with increased liver-related mortality and the development of hepatocellular carcinoma (HCC) [1,3,4]. Following the worldwide increase in NAFLD prevalence, NASH has become the third cause for liver transplantation in the United States in the setting of liver cirrhosis, and recent data suggests that NAFLD is a key factor associated with the increased incidence of HCC in Western countries [5,6].

The abundance of food, particularly high in fat, the increase in portion sizes, and consumption of beverages sweetened with sucrose or high-fructose corn syrup are all dietary factors that have been incriminated for the current rise in the incidence of obesity and the metabolic syndrome [7]. Features of the metabolic syndrome are epidemiologically closely linked to NAFLD; up to 61% and 28% of NAFLD subjects are obese and diabetic, respectively [1,8]. Crucially, the same dietary factors linked to the rise in obesity are also associated with the development of NAFLD [9]. High fructose consumption may increase the risk of NAFLD, although critical confounders, such as the level of physical activity and overall energy intake, have yet to be completely unraveled [10].

Despite the strong association between dietary factors and NAFLD, translational research in the field of NAFLD has been limited by the multitude of dietary models and the complex phenotype of the disease, both metabolically and in the liver. In addition, the relevance of individual animal models of the disease to the human phenotype has been questioned, both in the field of NAFLD and in other diseases [11,12]. One answer to this clinical conundrum may be to further leverage the vast quantity of -omics data (including genome, transcriptome, proteome, and methylome data, etc.) both in human subjects and in animal models of disease using the principles of “big data” to narrow the translational gap between the bench and the bedside [13,14]. In this review, we discuss some key topics of translational aspects of NAFLD research from the perspective of nutrition and dietary interventions.

## 2. Key Mechanisms Involved in the Pathogenesis of NAFLD

An influential hypothesis encompassing the pathogenesis of NASH was first encompassed by Day and colleagues as the “two-hit hypothesis”, where a first hit, steatosis, was followed by an additional insult, for example oxidative stress, for the development of steatohepatitis [15]. Nevertheless, growing evidence has shown that the pathogenesis of NASH requires multiple interrelated processes, including cytokine-mediated liver inflammation and injury, apoptosis mediated by free fatty acids, insulin resistance, and white adipose tissue dysfunction, which collectively lead to hepatic steatosis, inflammation, and ultimately fibrosis [16,17]. Importantly, the various histological and clinical stages of NAFLD are not only consequences of environmental or modifiable factors, such as diet, obesity, and insulin resistance, but are also tightly intertwined with genetic host factors, such as polymorphisms in the Patatin-Like Phospholipase Domain Containing 3 (PNPLA3) gene [18]. Nevertheless, it has to be said that the exact mechanisms underlying the development and progression of NAFLD to NASH remain unclear. In particular, human studies of the pathophysiology of NAFLD have been limited by the difficulties of unraveling the complex causes and consequences of NAFLD. In addition, as discussed below, the translation of animal models to human NAFLD remains limited as no single animal model recapitulates all subsets of the human disease.

## 3. Dietary Animal Models of NAFLD

Increasing recognition of the disease and additional efforts to elucidate its pathogenesis have led to an increase in animal models of NAFLD over the past years [19,20]. Recent overnutrition models of NAFLD have attempted to mimic the metabolic derangements seen in the disease as well as the hepatic histological changes. Below, we detail the most common animal models of NAFLD, focusing in particular on dietary models (Table 1).

### 3.1. High-Fat Diet (HFD)

The association between NAFLD and obesity led to the development of an HFD that matches modern Western diets. The first described high-fat diet, tested in male Sprague–Dawley rats by Lieber et al. (71% of energy from fat, 11% from carbohydrates, 18% from proteins) demonstrated the development of steatosis within three weeks associated with insulin resistance and increased markers of fibrogenesis [21]. Subsequently, similar results were reported in male C57BL/6 mice fed with an HFD for up to 16 weeks. The HFD group demonstrated an increase in body weight, the development of hepatic steatosis, hepatocyte ballooning, increased fasting serum glucose levels, and decreased adiponectin levels, suggesting hyperglycemia and insulin resistance [22]. Nevertheless, HFD seems to have a more pronounced effect in rats compared to mice, as less time is required to induce a more severe histological phenotype in rats. HFD can therefore replicate the altered metabolic parameters observed in human NAFLD, but the hepatic pathological outcome is not as severe.

### 3.2. Methionine- and Choline-Deficient (MCD) Diet

The MCD is one of the most popular dietary models of NASH. This lipogenic diet is generally rich in sucrose and fat (sucrose: 40% of energy; fat: 10% of energy) but is deficient in methionine and choline. Methionine and choline are essential for hepatic β oxidation and the production of very low-density lipoproteins (VLDL), and their deficiency leads to extensive hepatic inflammation as early as two weeks of feeding, as well as significant fibrosis after six weeks [23]. Although this model closely replicates the liver histological features of NAFLD and NASH, a major concern is that its metabolic features are very distinct from human NAFLD. Animals fed an MCD generally lose weight, have low fasting blood sugar, improved peripheral insulin sensitivity, low serum insulin and leptin levels, and unchanged or increased serum adiponectin levels as well as decreased blood triglycerides and cholesterol, contrasting strongly with the metabolic profile seen in humans [24,25]. One attempt to mitigate these differences has been to add MCD to *db*/*db* or *ob*/*ob* mice to better replicate the human metabolic and hepatic profile seen in NAFLD.

### 3.3. High-Cholesterol Diet

High-cholesterol diet is an important risk factor for the progression of hepatic inflammation in NAFLD both in animal models and in humans [26,27]. Nevertheless, although mice fed a high-cholesterol diet (1%) alone demonstrate increases in serum insulin levels, the increases in liver weight, triglyceride levels, and serum ALT levels are more modest [27]. Features of NASH are more manifest when a high-cholesterol diet is combined with an HFD or a dietary source of cholate [27]. For instance, mice fed an “atherogenic” diet consisting of 1.25% cholesterol, 7.5% cocoa butter, and 0.5% cholate over three weeks displayed increases of acute inflammation genes by the cholesterol component in the diet, and fibrogenesis genes by the cholate component [28]. Alternatively, mice fed a high-cholesterol combined with an HFD showed increased hepatic steatosis, inflammation, and perisinusoidal fibrosis, associated with adipose tissue inflammation, hypercholesterolemia, and obesity as well as a reduction in plasma adiponectin levels, compared to HFD or high-cholesterol diet alone [27].

### 3.4. High-Fructose Diet

Fructose consumption has been linked to obesity, dyslipidemia, and insulin resistance, and growing evidence suggests that fructose contributes to the development and severity of NAFLD [29]. Increasing quantities of dietary fructose come from sugar additives (most commonly sucrose and high-fructose corn syrup) in beverages and processed foods. In a study assessing C57BL/6 mice fed an HFD or a high-fat, high-fructose (HFHC) diet, the authors found that both the HFD and HFHC groups had increased body weight, body fat mass, fasting glucose, and were insulin-resistant compared with chow-fed mice. However, fibrogenesis, collagen deposition, hepatic oxidative stress, and plasma cholesterol were significantly increased in HFHC mice, suggesting that fructose may induce fibrogenesis through reactive oxygen species-induced mechanisms [30]. More recently, an HFD model with ad libitum consumption of glucose and fructose in a specific strain of mice was found to induce steatosis at four–eight weeks, NASH at 16–24 weeks, fibrosis after 16 weeks and spontaneous hepatocellular carcinoma [31].

### 3.5. Ketogenic Diet

A ketogenic diet has been shown to induce hepatic steatosis in rodents, although this is controversial in humans due to variations in macronutrients composition [32]. A ketogenic diet also induces hepatic inflammation and lipid accumulation in mice, while it reduces inflammation in white adipose tissue [33]. In a microscopic analysis, a ketogenic diet induced macrovesicular steatosis, parenchymal inflammatory foci, and features of regeneration, but without evidence of ballooning or fibrosis [34]. A ketogenic diet leads to hepatic steatosis in both short-term [35,36] and long-term feeding in mice [37]. In parallel with increased hepatic triglyceride content, biological markers such as AST and ALT are increased at least twofold [35]. Although controversial, a ketogenic diet has been shown to cause hepatic insulin resistance [35], which represents a key step towards the development of type 2 diabetes. In this case, hepatic insulin resistance was due to increased hepatic diacylglycerols, which are known to activate protein kinase Cε [38]. Indeed, triglycerides are usually considered inert regarding the development of insulin resistance, whereas diacylglycerols, but also ceramides, have been linked to the development of insulin resistance [39]. Overall, a ketogenic diet induces NAFLD in rodents, with some degree of inflammation, but does not induce more advanced stages pertaining to NASH, such as fibrosis. Moreover, although a ketogenic diet causes whole-body and hepatic insulin resistance, it does not induce weight gain. Indeed, mice fed a ketogenic diet gain less weight than mice fed regular chow, potentially secondary due to an increased energy expenditure [35]. Therefore, ketogenic diet does not represent an ideal model of metabolic syndrome associated with NAFLD/NASH.

### 3.6. Other Animal Dietary Models of NAFLD

The choline-deficient L-amino-defined (CDAA) diet is similar to the choline-deficient diet, but replaces proteins with a mixture of L-amino acids. C57BL/6 mice fed a CDAA diet developed liver injury that mimicked NASH features while inducing peripheral insulin resistance [40]. In addition, 40% of mice developed HCCs (100% of animals in a combined intraperitoneal CCl_4_-CDAA arm) at nine months of diet [40]. However, mice on this diet do not develop hepatic insulin resistance and long-term feeding of 20 weeks or more is required before liver fibrosis is observed [41,42].

Conjugated linoleic acid (CLA) refers to a mixture of positional and geometric isomers of linoleic acid. Sources of CLA include dairy products and meat from ruminant animals or those contained in synthetic products [43]. Although CLA diet has been associated with hepatic steatosis in animal models [44,45], its use as a valid animal model of NAFLD is limited due to the absence of the metabolic profile seen in humans and the wide interspecies variability of effect of CLA on liver histology [43,46].

### 3.7. Non-Dietary Animal Models of NAFLD

Numerous other models of NAFLD have been described. Genetic models of NAFLD include *db*/*db* mice, homozygous for a mutation in the leptin receptor leading to defective leptin signaling, *ob*/*ob* mice with autosomal recessive mutation in the leptin gene leading to mice that are grossly overweight, hyperphagic, hyperinsulinemic, hyperglycemic, and resistant to insulin. *Ob*/*ob* mice develop spontaneous liver steatosis but do not develop NASH unless secondary triggers are added [47]. Interestingly, unlike *db*/*db* mice, *ob*/*ob* mice are resistant to fibrosis, as leptin is an essential mediator of hepatic fibrosis [48].

A number of non-rodent animal models of NAFLD have also been reported. For example, dietary manipulation of the Ossabaw pig led to the development of the metabolic syndrome and features of NASH in this animal [49], while feeding Japanese white rabbits a diet supplemented with cholesterol and corn oil for nine months led to the development of features of NASH and advanced fibrosis [50]. However, there are practical challenges to the use of non-rodent animal models of NAFLD, such as higher costs and complexity of animal housing and less complete characterization of NAFLD and associated diseases in other species.

## 4. Selection of the Ideal Animal Model for Translational Research in NAFLD

The ideal animal model of NASH/NAFLD would encompass the metabolic features of the human disease, including obesity, dyslipidemia, insulin resistance, as well as replicate the liver histological findings found in patients. Unfortunately, the perfect human NASH model has not yet been identified or developed, although such a mouse model of NASH would greatly facilitate and encourage drug and biomarker discovery in the field [13]. Below, we discuss challenges and opportunities for the selection of animal models in the field of NAFLD.

In the framework of preclinical studies for therapeutic discovery, the value of animal models in general has been somewhat controversial [51]. Although animal models have significantly contributed to our understanding of diseases, they have been less successful for predicting accurate translation to predict effective treatment strategies. In a systematic review, Hackam et al. found that only one third of highly cited articles (defined as over 500 citations) translated at the level of human randomized trials [52]. Although a number of factors can explain these findings, a key factor is the limited external validity of numerous animal models to model complex multifactorial human diseases [51]. For example, in a systematic comparison of the genomic response between human inflammatory diseases and murine models, Seok et al. found a very poor correlation between genomic aberrations seen in the models and the corresponding human disease [12]. However, confusingly, after reevaluating the same gene expression datasets used in the previous study but focusing on genes whose expression levels were significantly changed in both humans and mice, Takao et al. found highly correlated gene expression changes and pathways in mice and humans [11]. These contrasting findings underline the huge remaining gap of knowledge limiting the full understanding of the relationship between animal models and the human disease.

One strategy to overcome this translational challenge is to adopt a multiscale setting including big data approaches combining clinical phenotype, genomic heterogeneity, transcriptomics, and metabolomics changes to identify the ideal NAFLD animal model for a given scientific question or to test a given drug. Unfortunately, the theoretical framework and robust cross-species technologies for this task are currently lacking; therefore, authors have preferred to rely on transcriptomics, or the systematic and global assessment of RNA expression in a given tissue as a reasonable proxy for such approaches. For example, the transcriptomic assessment of liver tissue in “severe” NAFLD fibrosis with advanced fibrosis compared to “mild” NAFLD with low levels of fibrosis found that metabolic and regenerative pathways were deregulated in severe NAFLD, and revealed an overlap among the gene expression patterns of severe NAFLD, cardiovascular disease, and cancer [53]. Interestingly, a 64-gene signature reproducibly differentiated severe NAFLD from mild NAFLD, and a 20-gene subset within this profile correlated with NAFLD severity, independent of other factors known to influence NAFLD progression, suggesting the possible translation to serum biomarkers and non-invasive markers of NAFLD severity. Another study assessing liver samples from morbidly obese subjects with all stages of NAFLD before and after bariatric surgery showed that NAFLD-associated methylation changes to be partially reversible pointing to treatment-induced epigenetic organ remodeling in humans [54]. Further integrating animal and human transcriptomic data, an NAFLD mouse model combining HFD with ad libitum consumption of glucose and fructose [31] showed by hepatic gene expression at 52 weeks of dietary intervention that the gene signature was concordant with a human liver cirrhosis signature [55], and that the HCC developed within this model were concordant with the gene expression of specific molecular subclasses seen in human HCC [56]. Teufel et al. further extended this approach by developing a platform for the selection of nine animal NAFLD models and their comparison to human liver disease through liver gene expression profiles and concluded that, at a pathway level, HFD was associated more closely to human NAFLD [14]. Table 2 summarizes further examples of the contribution of transcriptomics to preclinical therapeutic development in NAFLD using dietary NAFLD models.

## 5. Conclusions

NAFLD is a complex disease with an increasing epidemiology. Currently, no specific therapeutic alternative has been developed, partly due to the lack of robust reproducible dietary animal models of the disease. Novel big data approaches may help to bridge the translational gap for selecting dietary models of the disease for specific research questions or for testing specific drugs. It is likely that, in line with the worrying epidemiological expansion of the disease and rising interest from the industry, translational efforts aiming to address these issues will further develop in the future.

## Figures and Tables

**Table 1:** Features of the main dietary animal models of NAFLD.

Animal Model	Obesity	Insulin Resistance	Steatosis	Inflammation	Ballooning	Fibrosis
**HFD**	+	+	+	+/−	−	−
**MCD**	−	Hepatic IR only	+	+	+/−	+
**High-cholesterol**	−	Hepatic IR only	+	+	+	+
**High-fructose**	+	+	+	+	+/−	+
**Ketogenic**	−	+	+	+/−	−	−

HFD, high-fat diet; IR, insulin resistance; MCD, methionine- and choline-deficient diet; NAFLD, non-alcoholic fatty liver disease.

**Table 2:** Selected examples of contribution of transcriptomics to preclinical therapeutic discovery in NAFLD.

Animal Model of NAFLD	Technology and Assay	Research Context	Key Results	reference
Western style lard-rich diet in Sprague-Dawley rats	Transcriptomics: RNA-seq	Assessment of the effect of fish oil on a rat model of NASH by transcriptomics	Fish oil restored the expression of circardian clock-related genes, fatty acid genes, and inflammatory genes compared to the NASH rats without fish oil	[57]
Atherogenic-like diet (high fat, cholesterol and cholate) in C57BL/6J mice	Transcriptomics: microarray	Assessment of the effect of astaxanthin and vitamin E on a mouse model of NASH by transcriptomics	Assessment of differential effects of astaxanthin and vitamin E on the NASH model	[58]
HFD in C57BL/6J mice	Transcriptomics: RNA-seq	Assessment of the effect of vertical sleeve gastrectomy on a mouse model of NAFLD by transcriptomics	Regulatory regions and gene expression of lipid metabolism is altered by vertical sleeve gastrectomy	[59]
High fat, high sucrose and cholesterol diet or MCD diet in C57BL/6J mice	Transcriptomics: microarray	Identification of novel targets driving the transition to NASH and fibrosis by transcriptomic meta-analysis combining public human datasets with murine models	Dermatopontin expression was found increased in fibrosis, and reversal of fibrosis after gastric bypass correlated with decreased dermatopontin expression	[60]

NASH, nonalcoholic steatohepatitis.
